# Supplementary material for: Amyloid Fibrils of Stefin B Show Anisotropic Properties
Source: Int J Mol Sci. 2023 Feb 13;24(4):3737. doi: 10.3390/ijms24043737 (PMC9962164; doi:10.3390/ijms24043737)
Supplement: Supplementary file 1 [file ijms-24-03737-s001.zip › ijms-1872635-supplementary.pdf]

Supplementary Figure S1

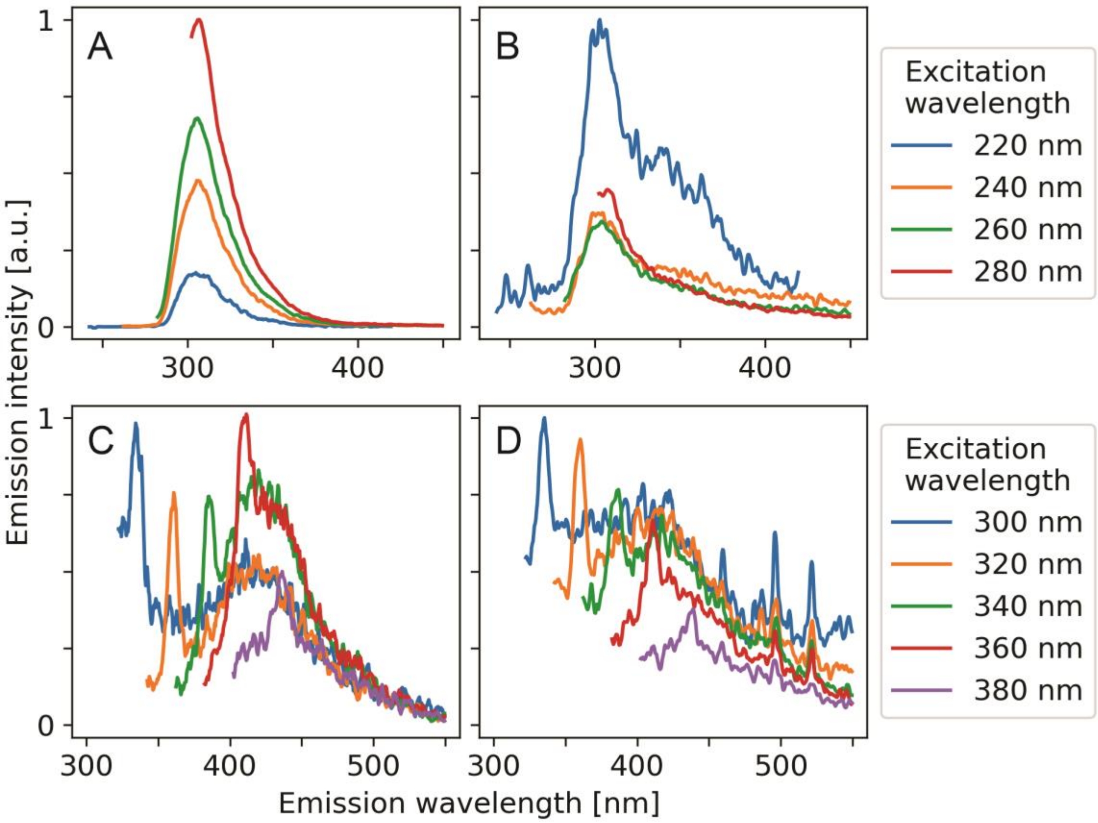

Legend to Suppl. Figure S1: **Fluorescence emission spectra** of the native protein in A) and C) and of amyloid fibrils in B) and D). In A) and B) are plotted fluorescence emission spectra from 250 to 450 nm excited from 220 to 280 nm, in steps of 20 nm. In C) and D) are plotted fluorescence emission spectra from 350 to 550 nm, excited from 300 to 380 nm, in steps of 20 nm.
